# Oligomeric Proanthocyanidins and Bamboo Leaf Flavonoids Improve the Quality of Bull Semen Cryopreservation

**DOI:** 10.3390/molecules27031144

**Published:** 2022-02-08

**Authors:** Hongtao Wang, Ping Lu, Zhiqiang Li, Chongshan Yuan, Hongyu Liu, Jing Zhao, Wenfa Lu, Jun Wang

**Affiliations:** 1 Joint Laboratory of Modern Agricultural Technology International Cooperation, Ministry of Education, Jilin Agricultural University, Changchun 130118, China; hongtao2021@yeah.net (H.W.); luping100419@163.com (P.L.); lizhiqiangsky@126.com (Z.L.); 18844146800@163.com (C.Y.); jlndlhy0133@163.com (H.L.); 2 Key Lab of Animal Production, Product Quality and Security, Ministry of Education, Jilin Agricultural University, Changchun 130118, China; 3 College of Animal Science and Technology, Jilin Agricultural University, 2888 Xincheng Street, Changchun 130118, China

**Keywords:** oligomeric proanthocyanidins, antioxidant enzymes, Simmental bull, semen freezing, sperm analysis

## Abstract

It is important to inhibit oxidative stress to maintain sperm motility during cryopreservation. The present study was performed to investigate the effects of supplementing oligomeric proanthocyanidins (OPC) and bamboo leaf flavonoids (BLF) or their combination as an extender for Simmental bull semen freezing. OPC, BLF, or their combination were added to the frozen diluent of bovine semen. Afterwards, computer-assisted semen analysis (CASA), detection of membrane functionality, acrosome integrity, mitochondrial integrity, CAT, SOD, GSH-PX, MDA, and ROS were conducted. The results showed that adding 50 mg/L OPC or 4 mg/L BLF could improve the quality of frozen sperm. Compared with 50 mg/L OPC alone, the combination of 50mg/L OPC and 2 mg/L BLF significantly increased the kinematic parameters of sperm, and sperm CAT, GSH-PX and SOD levels (*p* < 0.05), whereas the MDA of sperm was decreased (*p* < 0.05). These results indicated that compared to the addition of 50 mg/L OPC alone, a combination of 50 mg/L OPC and 2 mg/L BLF could further improve the quality of frozen semen. The results could provide theoretical data support for the development of a new protective agent and are significant for the cryopreservation of bovine semen in the future.

## 1. Introduction

The cryopreservation of mammal semen is accompanied by the generation of oxidative stress, which could decrease sperm motility and induce DNA damage. Oxidative stress is one of the main reasons for the decrease in the success rate of artificial insemination [1]. In addition, mammalian sperms have relatively high polyunsaturated fatty acids in their plasma membranes (PUFA), which is why mammalian sperm are more susceptible to oxidative stress [2]. Oxidative stress is mainly caused by the imbalance of reactive oxygen species (ROS) and various antioxidant enzymes in semen, such as superoxide dismutase (SOD), glutathione peroxidase (GSH-PX), and catalase (CAT). Spermatozoa are protected by various antioxidants and antioxidant enzymes in the seminal plasma or in spermatozoa itself to prevent oxidative damage [3]. However, the antioxidant capacity of mature spermatozoa is low and not sufficient to cope with ROS produced during oxidative stress. In order to alleviate the oxidative stress caused by this imbalance, adding antioxidants to the diluent has become a necessary means [4]. Adding antioxidants has been found to improve semen quality in many species [5,6,7,8,9].

As a kind of natural polyphenol, oligomeric proanthocyanidins (OPC) exist widely in nature [10]. OPC have been found in some studies to protect cells from H_2_O_2_-induced oxidative stress [11], and also has many biological activities such as inhibiting apoptosis [12], anticancer [13], anti-aging [14], and antioxidants [15]. In terms of antioxidants, OPC could reduce blood pressure by relieving the oxidative stress level of mice [16]. OPC could also improve the antioxidant capacity of pig semen to improve the pregnancy rate of sows and the survival rate of piglets [17], because different antioxidants have a synergistic effect on the removal of ROS. As a natural plant extract, bamboo leaf flavonoids (BLF) have a strong antioxidant ability [18], in addition to having a strong antioxidant capacity. In addition, BLF have a very strong performance in immune regulation [19], as well as anti-inflammatory [20], anti-depression [21], and lipid-lowering properties [22]. In recent studies, it was found that the addition of BLF can reduce oxidative stress of cells by regulating ROS production and activities of various antioxidant enzymes [23]. A large number of studies have shown that OPC and BLF have a strong antioxidant ability, but they have not been used in cryopreservation of bovine semen.

Therefore, the purpose of this study was to research the effects of OPC and BLF on kinematic parameters, plasma membranes, acrosome integrity, antioxidant enzyme content, ROS and MDA levels of bovine semen freezing, observe whether the combination of OPC and BLF could further improve the quality of semen, and explore whether adding OPC and BLF could improve the quality of freeze-thawed bull semen.

## 2. Results

### 2.1. Effects of Different Concentrations OPC and BLF Separately on Parameters of Bovine Semen after Thawing

#### 2.1.1. Effects of Different Concentrations of OPC addition on Kinematic Parameters of Frozen Bovine Semen

As shown in Table 1, adding 0, 10, 30, 50, and 70 mg/L OPC could improve the kinematic parameters of frozen semen. The effect of 50 mg/L OPC on the VCL, VAP, VSL kinematic parameters is higher than other concentrations (*p* < 0.05).

#### 2.1.2. Effects of Different Concentrations of BLF addition on Kinematic Parameters of Frozen Bovine Semen

As shown in Table 2, adding 0, 2, 4, 6, and 8 mg/L BLF could improve the kinematic parameters of frozen semen. The effect of 4 mg/L BLF on the VCL, VAP, and VSL kinematic parameters is higher than other concentrations (*p* < 0.05).

#### 2.1.3. Effects of Different Concentrations of OPC on the Integrity of the Plasma Membrane, Acrosome, and Total Motility after Thawing of Bovine Semen

**Figure 1:**
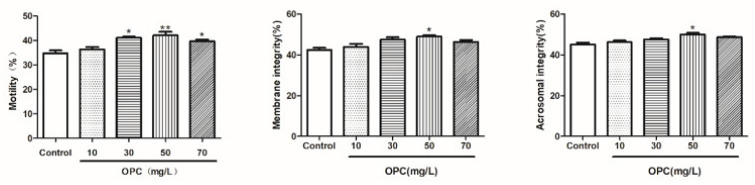
Effects of different concentrations of OPC on the integrity of the plasma membrane, acrosome, and total motility after thawing of bovine semen, * indicates a significant difference compared to that of the control group (* *p* < 0.05. ** *p* < 0.01).

Results of the plasma membrane, acrosome, and total motility after the semen cryopreservation in 0, 10, 30, 50, and 70 mg/L of OPC are depicted in Figure 1. The total motility in the OPC 50 mg/L groups is greater (*p* < 0.01) than that in the different concentrations. The integrity of the plasma membrane and acrosome in the OPC 50 mg/L groups was greater (*p* < 0.05) than that in the different concentrations.

#### 2.1.4. Effects of Different Concentrations of BLF on the Integrity of the Plasma Membrane, Acrosome, and Total Motility after Thawing of Bovine Semen

**Figure 2:**
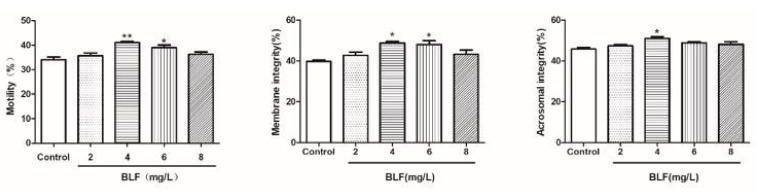
Effects of different concentrations of BLF on the integrity of the plasma membrane, acrosome, and total motility after thawing of bovine semen, * indicates a significant difference compared to that of the control group (* *p* < 0.05. ** *p* < 0.01).

Results of the plasma membrane, acrosome, and total motility after the semen cryopreservation in 0, 2, 4, 6, and 8 mg/L of BLF are depicted in Figure 2. The total motility in the BLF 4 mg/L groups is greater (*p* < 0.01) than that in the different concentrations. The integrity of the plasma membrane and acrosome in the BLF 4 mg/L groups was greater (*p* < 0.05) than that in the different concentrations.

#### 2.1.5. Effects of Different Concentrations of OPC on the Antioxidant Enzyme Activity of Bovine Semen after Thawing

**Figure 3:**
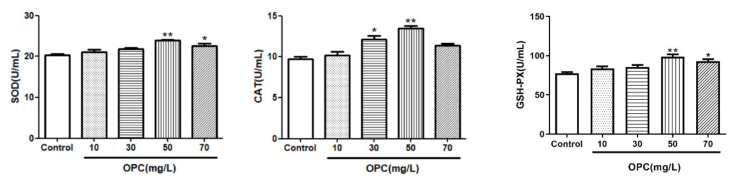
Effects of different concentrations of OPC on the antioxidant enzyme activity of bull semen after thawing, * indicates a significant difference compared to that of the control group (* *p* < 0.05. ** *p* < 0.01).

Results of SOD, CAT, and GSH-PX after the semen cryopreservation in 0, 10, 30, 50, and 70 mg/L of OPC are depicted in Figure 3. The SOD, CAT, and GSH-PX in the OPC 50 mg/L groups were greater (*p* < 0.01) than that in the different concentrations.

#### 2.1.6. Effects of Different Concentrations of BLF on the Antioxidant Enzyme Activity of Bovine Semen after Thawing

**Figure 4:**
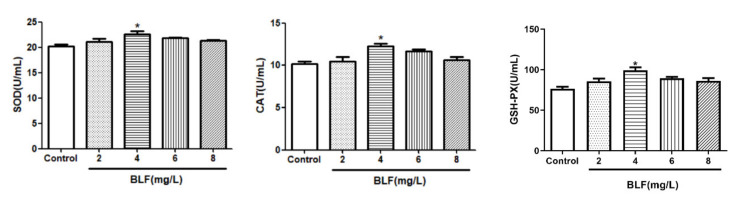
Effects of different concentrations of BLF on the antioxidant enzyme activity of bull semen after thawing, * indicates a significant difference compared to that of the control group (* *p* < 0.05. ** *p* < 0.01).

Results of SOD, CAT, and GSH-PX after the semen cryopreservation in 0, 2, 4, 6, and 8 mg/L of BLF are depicted in Figure 4. The SOD, CAT and GSH-PX in the BLF 4 mg/L groups were greater (*p* < 0.05) than that in the different concentrations.

#### 2.1.7. Effects of Different Concentrations of OPC on the Oxidation Products of Bovine Semen after Thawing

**Figure 5:**
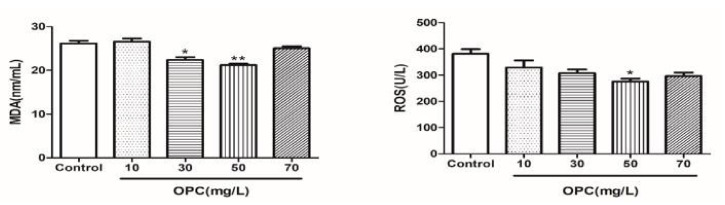
Effects of different concentrations of OPC on the oxidation products of cattle semen after thawing, * indicates a significant difference compared to that of the control group (* *p* < 0.05. ** *p* < 0.01).

Results of MDA and ROS after the sperm cryopreservation in 0, 10, 30, 50, and 70 mg/L of OPC are depicted in Figure 5. The MDA in the OPC 50 mg/L groups were lower (*p* < 0.01) than that in the different concentrations. The ROS in the OPC 50 mg/L groups were lower (*p* < 0.05) than that in the different concentrations.

#### 2.1.8. Effects of Different Concentrations of BLF on the Oxidation Products of Bovine Semen after Thawing

**Figure 6:**
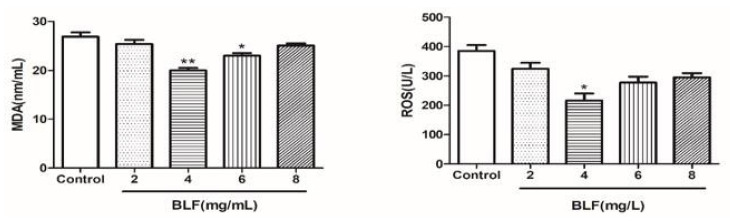
Effects of different concentrations of BLF on the oxidation products of cattle semen after thawing, * indicates a significant difference compared to that of the control group (* *p* < 0.05. ** *p* < 0.01).

The results of MDA and ROS after the sperm cryopreservation in 0, 2, 4, 6, and 8 mg/L of BLF are depicted in Figure 6. The MDA in the BLF 4 mg/L groups were lower (*p* < 0.01) than that in the different concentrations. The ROS in the BLF 4 mg/L groups were lower (*p* < 0.05) than that in the different concentrations.

### 2.2. Effects of Combined Addition of OPC and BLF on Parameters of Semen after Thawing

#### 2.2.1. Effects of Combination of 50 mg/L OPC and Different Concentration of BLF on Kinematic Parameters of Frozen Bovine Semen

As shown in Table 3, the combination addition further improved the kinematic parameters of frozen semen compared to that with 50 mg/L OPC alone. A combination of 50 mg/L OPC and 2 mg /L BLF significantly improved BCF, VAP, VCL, VSL, and ALH kinematic parameters (*p* < 0.05).

#### 2.2.2. Effects of Combination of 50 mg/L OPC and Different Concentration of BLF on the Plasma Membrane, Acrosome, and Total Motility of Bovine Semen

**Figure 7:**
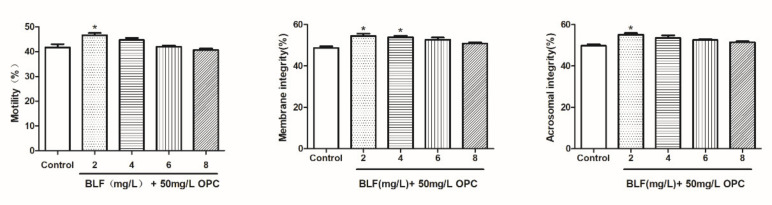
Effects of the combined use of OPC and BLF on the plasma membrane, acrosome, and total motility of bovine semen, * indicates a significant difference compared to that of the control group (control group is 50mg/L OPC) (* *p* < 0.05).

As shown in Figure 7, the combination addition further improved the total motility, sperm plasma membrane integrity, and acrosome integrity of frozen semen compared to that with 50 mg/L OPC alone. A combination of 50 mg/L OPC and 2 mg /L BLF significantly improved total motility, sperm plasma membrane integrity, and acrosome integrity of semen (*p* < 0.05).

#### 2.2.3. Effects of Combination of 50 mg/L OPC and Different Concentration of BLF on the on the Antioxidant Enzyme Activity of Bovine Semen after Thawing

**Figure 8:**
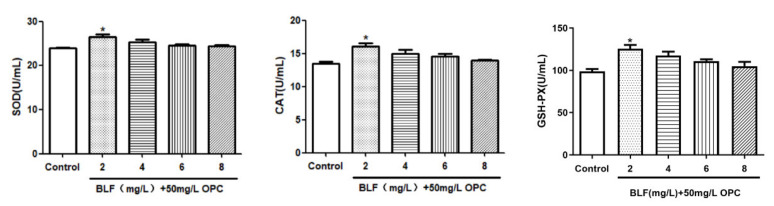
Effects of the combined use of OPC and BLF on the on the antioxidant enzyme activity of bull semen after thawing, * indicates a significant difference compared to that of the control group (control group is 50 mg/L OPC) (* *p* < 0.05).

As shown in Figure 8, the combination addition further improved the SOD, CAT, and GSH-PX of frozen semen compared to that with 50 mg/L OPC alone. A combination of 50 mg/L OPC and 2mg /L BLF significantly improved SOD, CAT and GSH-PX in semen (*p* < 0.05).

#### 2.2.4. Effects of Combination of 50 mg/L OPC and Different Concentration of BLF on the Oxidation Products of Bovine Semen after Thawing

**Figure 9:**
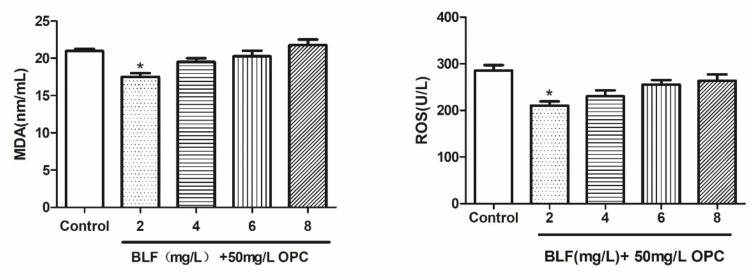
Effect of the combined use of OPC and BLF on the oxidation products of cattle semen after thawing, * indicates a significant difference compared to that of the control group (control group is 50mg/L OPC) (* *p* < 0.05).

As shown in Figure 9, the combination addition further reduced the ROS and MDA of frozen semen compared to that with 50 mg/L OPC alone. A combination of 50mg/L OPC and 2 mg /L BLF significantly reduced ROS and MDA in semen (*p* < 0.05). 

## 3. Materials and Methods

### 3.1. Chemicals

In this study, OPC was acquired from Shenzhen Zhenqiang Biological Technology Co., LTD. BLF was acquired from Hubei Yongkuo Technology Co., LTD (WuHan, China).

### 3.2. Experimental Design

In this study, pooled semen was extended using a Tris extender with different levels of OPC (0, 10, 30, 50, and 70 mg/L) and BLF (0, 2, 4, 6, and 8 mg/L) or a combination of OPC and BLF (50 mg/L OPC, 50mg/L OPC + 2 mg/L, 50 mg/L OPC + 4 mg/L, 50 mg/L OPC + 6 mg/L, and 50 mg/L OPC + 8mg/L). Each experiment was repeated at least three times.

### 3.3. Animals

This study was conducted at Jilin Agricultural University in P.R. China and approved by the Experimental Animal Welfare and Ethics Committee of Jilin Agricultural University (number is 20200803002). Four bulls (4 years old) were used for semen collection. The uniformity of feed, housing, and light conditions were ensured. The bulls had free access to water and salt, and no additional antioxidants were present in the feed. The bull basal diet composition is shown in Table 4, and the bull nutritional indicators in Table 5.

### 3.4. Bull Semen Collection

Semen samples were collected using an artificial vagina twice per week per bull; the samples were collected for 12 weeks. The criteria for cryopreservation were as follows. Semen samples were sent to the laboratory within 30 min. The semen was held in a water bath at 37 °C while the sperm concentration was estimated using a calibrated spectrophotometer and the motility of sperm was subjectively evaluated using microscopy at a concentration of at least 1 × 10^9^ spermatozoa/mL, sperm motility ≥ 70%, and abnormality ≤ 15%. Healthy ejaculates were used in the experiments. After the initial assessment, the semen samples were mixed to eliminate individual differences.

### 3.5. Basic Extender

The basic extender was slightly modified from that described by Tarig et al. [24]. The basic ingredients are 1.1 g glucose, 1.48 g citric acid, 2.42 g tris, 0.06 g penicillin sodium, and 0.1 g streptomycin sulfate. All the chemicals were placed into a capacity bottle and double evaporative water was used to stabilize the capacity to 100 mL, a magnetic stirrer was used to stir for 2 h, then 20% egg yolk and 3% glycerin were added, and stirred again for 2 h to completely dissolve. The basic diluent was divided evenly and OPC or BLF of different concentrations were added.

### 3.6. Semen Processing

After the quality evaluation, the samples were diluted and incubated in a water bath at 37 °C for 30 min for the full absorption of OPC and BLF by the sperm membrane. After that, the samples were packed in 0.25 mL straws (IMV, L’Aigle, France) with 8 × 10^6^ sperm/straws, then cooled from 37 °C to 4 °C for 2 h as previously described, and subsequently cooled for approximately 8 min from 4 °C to −140 °C by a turbo freezer (Minitubue, Germany). After that, the straws were transferred to a liquid nitrogen tank (−196 °C) and stored for a long time before detection.

### 3.7. Evaluation of Post-Thawed Sperm

#### 3.7.1. Computer Assisted Semen Analysis

The kinematic parameters of sperm were analyzed using a sperm analyzer (developed jointly by Hamilton and IMV, IVOS II, 10871, 6 October 2017). Two straws were thawed by immersion in a water bath at 37 °C for 30 s. Each sample was analyzed at least three times. Five microliters were detected for each straw, and four fields were randomly examined. Average path velocity (VPA), straight line velocity (VSL), curvilinear velocity (VCL), amplitude of lateral head displacement (ALH), beat/cross frequency (BCF), and linearity (LIN) were recorded.

#### 3.7.2. Acrosome Integrity

The sperm acrosome integrity was slightly modified from that described by Masoudi et al. [25]. The sample was centrifuged, and the resultant sperm pellet was obtained. It was then equilibrated in 96% ethanol for 10 minutes. Afterwards, the sperm was placed on a glass slide and fluorescein-isothiocyanate-conjugated pea lectin (PSA-FITC) (Sigma-Aldrich, St. Louis, MO, USA) was added. Slides were incubated for 20 min, and glycerol was added to fix the sperm on the slide. At least 5 fields of view through a fluorescence microscope were observed, with a minimum of 200 sperm in each field, and the percentage of sperm with green fluorescence on the sperm head to the total number was calculated

#### 3.7.3. Plasma Membrane Integrity

The method of measuring sperm acrosome integrity was slightly modified according to the method in the article published by R.A. Harrison et al. [26]. Using the double-staining method of carboxy fluorescein diacetate (CFDA) (Andy Forno Biotechnology (Wuhan) Co., Ltd., Wuhan, China) and propidium iodide (PI) (Coolaber), 0.46 mg CFDA was dissolved in 1 mL dimethyl sulfoxide and 0.5 mg PI was dissolved in 1 mL normal saline. It was stored at −20 °C and protected from light. Afterwards, 20 μL CFDA and 10 μL PI were dissolved in 1 mL of PBS with a pH of 7.4 and 0.01 mol/L. A 20 μL semen sample was then taken and 80 μL staining solution was added to it. The cells were incubated at 37 °C for 10 min and washed with PBS. After washing, 10 μL was taken and observed under a fluorescence microscope, with a minimum of 200 sperm in each field, and the percentage of sperm with green fluorescence on the sperm head to the total number was calculated.

#### 3.7.4. Endogenous Antioxidant Indices Detection in the Frozen–Thawed Semen

The determination of various endogenous antioxidant enzymes (superoxide dismutase, glutathione peroxidase, and catalase) was carried using an enzyme-linked immunoassay kit (Shanghai Enzyme Biotechnology Co., Ltd., Shanghai, China), according to the manufacturer’s instructions. The sample tested was mixed with the sample diluent. After incubation, the enzyme-labeling reagent and stop solution were added in sequence. The resulting mixture was then placed into the microplate reader for analysis.

#### 3.7.5. MDA and ROS Concentration Determination in Post-Thawed Semen

A bull MDA and ROS ELISA assay kit was used to determine the concentrations of MDA and ROS (Shanghai Enzyme Biotechnology Co. Ltd, Shanghai, China). The bovine semen was centrifuged repeatedly to destroy the sperm structure. Next, 40 μL of diluent was added to the sample, and placed on a shaker to mix the semen and dilution thoroughly. It was then incubated in a 37 °C incubator for 30 min, washed with washing solution five times, then 50 μL of enzyme-labeled reagent was added, again incubated in a 37 °C incubator for 30 min, and washed 5 times after equilibration. Two kinds of diluent shake were then added and mixed thoroughly. The entire procedure was performed for 10 min at 37 °C in the dark. After adding the stop solution, the absorbance was measured with a microplate reader at a wavelength of 450 nm, and the contents of MDA and ROS were calculated according to the standard curve. 

### 3.8. Statistical Analyses

The data were analyzed using GraphPad Prism 5 software. Unless otherwise stated, significance was set at *p* < 0.05. The result is expressed as mean ± SEM. A one-way analysis of variance was performed to evaluate sperm motility, acrosome integrity, plasma membrane integrity, antioxidant enzyme content, ROS level, oxidation product, malondialdehyde content, and mitochondrial membrane potential.

## 4. Discussion

In order to improve the quality of cryopreservation semen and reduce the effect of oxidative stress, adding antioxidants to cryopreservation diluents has become an important method [27]. Our results showed that adding 50 mg/L OPC or 4 mg/L BLF could improve the quality of frozen semen. Measurements of 50 mg/L OPC and 2 mg/L BLF or 4 mg/L BLF and 30 mg/L OPC were added in combination, and the quality of semen was further improved when compared to adding them separately.

Sperm motility is the main factor affecting reproductive efficiency. Furthermore, the kinematic parameters of sperm may be reliable indicators to evaluate the success of cryopreservation and fertilization [28]. In this study, it was found that adding OPC alone could significantly improve sperm motility and kinematic parameters after freezing. This suggests that OPC may act as an antioxidant to relieve sperm malformation rates and may improve pregnancy rates by improving sperm motility. The integrity of the plasma membrane and acrosome is an important factor in whether sperm could be inseminated or not, and antioxidants in the diluent have the ability to protect sperm, help protect sperm motility, plasma membrane, acrosome integrity, and fertilization ability [29,30]. In our research, it was found that after adding OPC, the sperm plasma membrane and acrosome intact rate were significantly improved, which may help sperm to pass through the zona pellucida and vitelline membrane and increase the pregnancy rate. MDA overproduction is one of the main causes of spermatozoa LPO. Studies have found that adding OPC can alleviate atherosclerosis in rats by increasing the content of SOD and GSH-PX and decreasing the level of MDA [31]. Similar results were also obtained in this study. After adding OPC, SOD, and GSH-PX contents in cryopreservation, semen was significantly increased, whereas the MDA level was significantly decreased. Excessive ROS is one of the main factors leading to oxidative stress of sperm, the imbalance between ROS, and various antioxidant enzymes in semen will result in decreased sperm motility, damaged morphology, and increased malformation rate [32,33,34]. In recent studies, it has been found that adding OPC could reduce the ROS level in boar semen and improve the total antioxidant capacity (T-AOC) [17]. This is consistent with our results. When OPC was added, the ROS level in semen was significantly reduced, and the antioxidant enzyme content, such as CAT, was significantly increased. This suggests that OPC may improve the sperm morphological parameters by increasing the level of various antioxidant enzymes in semen and decreasing the contents of MDA and ROS. The imbalance between the antioxidant enzyme content and ROS in semen is one of the main factors leading to oxidative stress, so adding antioxidants to diluent is necessary. Studies have found that add BLF could increase the production of antioxidant enzymes and mitochondrial membrane potential, and reduce oxidative stress and ROS production [23]. In our study, through the addition of BLF, the levels of various antioxidant enzymes, CAT, SOD and GSH-PX, were significantly improved, and there was also a significant reduction in ROS levels. Cryopreservation of semen could cause severe freezing damage. Freezing damage is often accompanied by inflammation, which often leads to the appearance of abnormal sperm. Recent studies have shown that BLF could reduce the release of inflammatory factors to improve inflammatory injury [21]. We observed through the sperm analyzer that the addition of BLF could significantly improve the abnormal motility of sperm. The levels of VSL, LIN, and VAP in sperm were also significantly improved, which may be due to BLF mitigating the release of inflammatory factors. Since the current cryopreservation process of semen is mostly carried out in an open environment, this could cause semen to be exposed to the air, causing bacterial contamination, and resulting in poor semen quality. It has been found that BLF could improve the immune function of chickens by improving the microflora [19]. This is consistent with our findings. Our research also found that BLF could improve the vitality of semen after thawing and other indicators. This may be due to the various biological activities of BLF.

Some studies have shown that different antioxidants have a synergistic effect on the removal of active oxygen. This is because they have different binding speeds with active free radicals and different ways of action [35]. On combining OPC and BLF, it was observed that the combination of two antioxidants could improve the quality of semen without other side effects. In addition, adding these substances could improve the vitality after freezing; however, this does not imply that they can improve the fertilization ability because there is no measurement of conception rate and other indicators, which is a limitation of this project.

In conclusion, adding 50 mg/L OPC or 4mg/L BLF could significantly improve the kinematic parameters of sperm, acrosome integrity, and plasma membrane integrity of bovine semen. However, a combination of 50mg/L OPC and 2 mg/L BLF could further improve the kinematic parameters of sperm, and the integrity of the acrosome, plasma membrane. These results may be due to the addition of OPC, and BLF alleviating the levels of ROS and MDA, as well as an increasing the enzymatic activity of CAT, SOD, and GSH-PX.

## Figures and Tables

**Table 1:** Kinematic parameters for computer-aided sperm motility analysis after adding OPC.

OPC (mg/L)	LIN (%)	BCF (Hz)	VCL (μm/s)	VAP (μm/s)	VSL (μm/s)	ALH (μm/s)
0	63.78 ± 1.55	19.45 ± 1.67	123.18 ± 1.08 ^a^	96.31 ± 3.91 ^a^	78.56 ± 3.28 ^a^	1.62 ± 0.08
10	63.66 ± 1.48	20.75 ± 2.66	124.80 ± 2.12 ^ab^	99.33 ± 1.87 ^ab^	79.45 ± 1.11 ^ac^	1.72 ± 0.07
30	67.06 ± 2.71	19.59 ± 2.49	125.19 ± 2.85 ^ab^	99.83 ± 1.66 ^ab^	83.96 ± 2.92 ^ab^	1.75 ± 0.12
50	65.56 ± 1.99	20.06 ± 1.53	130.61 ± 2.45 ^b^	104.67 ± 3.52 ^b^	85.63 ± 3.84 ^b^	1.64 ± 0.09
70	66.63 ± 2.21	19.51 ± 2.01	126.95 ± 2.53 ^ab^	98.86 ± 2.36 ^ab^	84.59 ± 2.81 ^bc^	1.60 ± 0.08

Abbreviations: Mean ± SEM of average path velocity (VAP), straight line velocity (VSL), curvilinear velocity (VCL), amplitude of lateral head displacement (ALH), beat/cross frequency (BCF), and linearity (LIN). Different lowercase letters in the same column of data indicate significant differences (*p* < 0.05), and the same lowercase letters indicate insignificant differences (*p* > 0.05).

**Table 2:** Kinematic parameters for computer-aided sperm motility analysis after adding BLF.

BLF (mg/L)	LIN (%)	BCF (Hz)	VCL (μm/s)	VAP (μm/s)	VSL (μm/s)	ALH (μm/s)
0	62.14 ± 1.39	21.09 ± 1.32	121.97 ± 1.98 ^a^	95.56 ± 1.43 ^a^	75.79 ± 2.62 ^a^	1.62 ± 0.04
2	64.52 ± 1.10	20.03 ± 1.81	123.73 ± 2.95 ^a^	98.91 ± 2.12 ^ab^	79.84 ± 2.96 ^ab^	1.68 ± 0.07
4	63.90 ± 2.15	19.17 ± 1.17	129.24 ± 3.54 ^b^	103.38 ± 2.22 ^b^	82.59 ± 2.32 ^b^	1.71 ± 0.08
6	65.49 ± 1.52	19.86 ± 1.61	124.68 ± 2.77 ^ab^	102.43 ± 2.82 ^b^	81.65 ± 1.23 ^b^	1.68 ± 0.07
8	65.85 ± 2.26	20.15 ± 1.86	122.16 ± 1.79 ^a^	100.37 ± 2.66 ^ab^	80.45 ± 2.18 ^ab^	1.67 ± 0.08

Abbreviations: Mean ± SEM of average path velocity (VAP), straight line velocity (VSL), curvilinear velocity (VCL), amplitude of lateral head displacement (ALH), beat/cross frequency (BCF), and linearity (LIN). Different lowercase letters in the same column of data indicate significant differences (*p* < 0.05), and the same lowercase letters indicate insignificant differences (*p* > 0.05).

**Table 3:** Kinematic parameters of computer-aided sperm motility analysis after adding OPC and BLF.

OPC (mg/L)/BLF (mg/L)	LIN (%)	BCF (Hz)	VCL (μm/s)	VAP (μm/s)	VSL (μm/s)	ALH (μm/s)
OPC50	65.71 ± 1.99	20.13 ± 1.53 ^a^	130.32 ± 2.45 ^a^	104.41 ± 3.52 ^a^	85.35 ± 3.84 ^a^	1.63 ± 0.09 ^a^
OPC50 + BLF2	65.89 ± 1.18	24.60 ± 1.02 ^b^	138.35 ± 2.79 ^b^	110.65 ± 1.84 ^b^	91.16 ± 2.31 ^b^	1.71 ± 0.08 ^b^
OPC50 + BLF4	64.51 ± 2.75	23.91 ± 1.17 ^ab^	137.45 ± 1.65 ^b^	107.77 ± 3.09 ^ab^	88.67 ± 2.96 ^ab^	1.70 ± 0.02 ^b^
OPC50 + BLF6	66.52 ± 1.23	22.61 ± 1.79 ^ab^	133.02 ± 1.02 ^ab^	106.11 ± 3.45 ^ab^	88.49 ± 1.68 ^ab^	1.68 ± 0.07 ^ab^
OPC50 + BLF8	64.84 ± 2.84	21.73 ± 1.06 ^ab^	133.01 ± 1.09 ^ab^	105.74 ± 3.61 ^ab^	86.25 ± 3.15 ^ab^	1.67 ± 0.06 ^ab^

Abbreviations: Mean ± SEM of average path velocity (VAP), straight line velocity (VSL), curvilinear velocity (VCL), amplitude of lateral head displacement (ALH), beat/cross frequency (BCF), and linearity (LIN). Different lowercase letters in the same column of data indicate significant differences (*p* < 0.05), and the same lowercase letters indicate insignificant differences (*p* > 0.05).

**Table 4:** Bull basal diet composition.

Ingredient	Dosage
corn	45%
soybean cake	32%
wheat bran	5%
rice bran meal	5%
soybean germ meal	6%
molasses	2%
bull special premix	5%

**Table 5:** Bull nutritional indicators.

Ingredient	Dosage
crude protein	20%
moisture	13%
crude ash	7%
calcium	0.6–0.7%
total phosphorus	0.6–0.7%
sodium chloride	0.8–1%

## Data Availability

Not applicable.

## Sample Availability

Samples of the compounds are not available from the authors.
